# Aspirational leaders help us change: Ingroup prototypicality enables effective group psychotherapy leadership

**DOI:** 10.1111/bjc.12406

**Published:** 2022-12-02

**Authors:** Alysia M. Robertson, Tegan Cruwys, Mark Stevens, Michael J. Platow

**Affiliations:** ^1^ Research School of Psychology, The Australian National University Canberra ACT Australia

**Keywords:** group norms, group psychotherapy, group psychotherapy leadership, ingroup prototypicality, normative change

## Abstract

**Objectives:**

Research suggests that leaders are effective when they are ingroup prototypical (represent the identity of the group they seek to lead). However, it is unclear whether leaders should represent the group's *current* identity (“who we *are*”) or *aspired* identity (“who we *want to be*”). This study investigated which of these forms of prototypicality best predicted leadership effectiveness in group psychotherapy.

**Design:**

Longitudinal study.

**Methods:**

519 questionnaire responses were obtained from 112 women attending a four‐session body acceptance program. Focal measures included participant ratings of how often they thought their psychotherapy leaders and fellow group members would (a) engage in dieting thoughts and behaviours and (b) approve of dieting. Given the program's body acceptance focus, leader prototypicality was conceptualized as the difference between participants' perceptions of how often their leaders, versus group members, would diet at the start of therapy. Leadership effectiveness was conceptualized as reductions in perceived group approval of dieting across therapy. Two therapeutic outcomes were considered: body satisfaction and dieting intentions.

**Results:**

A mixed‐effects repeated measures analysis indicated that group approval of dieting decreased more rapidly when participants perceived their leaders to be aspirational (thought that group leaders dieted *less frequently* than group members) than when they perceived them to be exemplary (thought that group leaders dieted *as frequently* as group members). Changes in group approval of dieting mediated the relationship between leader prototypicality and improved therapeutic outcomes.

**Conclusions:**

Findings suggest that group psychotherapy leaders may increase their effectiveness by striving to embody their group's aspired identity.

**Practitioner Points**

- Clinicians may be more effective when they are perceived to represent the aspirational goals of a psychotherapy group, rather than to represent the group's current state.
- The way that participants of therapy groups view their therapists may influence how the norms of the group change over the course of therapy.
- Practical strategies for clinicians to demonstrate how they represent a group's aspirational identity could be integrated into clinician training to enhance psychotherapy effectiveness.

## BACKGROUND

Group psychotherapy has been increasingly recognized as an efficient and cost‐effective alternative to individual psychotherapy (Cuijpers et al., 2008; Tucker & Oei, 2007). However, delivering effective group psychotherapy sessions can be challenging for therapists, not least because doing so requires different skills (e.g., managing group processes) to those required to deliver effective individual psychotherapy sessions (Yalom & Leszcz, 2020). Despite this, empirical research seeking to describe how group psychotherapy leaders can maximize their effectiveness has been limited. Indeed, research in group psychotherapy contexts has rarely conceptualized those delivering group psychotherapy sessions as *leaders* and has thus failed to capitalize on the considerable insights regarding the bases of effective leadership that are offered by leadership theories (which have been developed, and often extensively tested, in other domains). The present study sought to advance understanding of how group psychotherapy leaders can maximize their effectiveness by investigating the benefits associated with group leaders engaging in behaviours consistent with the *social identity theory of leadership* (Haslam et al., 2020; Hogg, 2001; for recent applications in the context of individual psychotherapy see Lee et al., 2021; Platow et al., 2020).

### Theoretical framework: social identity theory of leadership

The social identity approach recognizes people's capacity to define themselves both in terms of their personal identity (as “I” and “me”) and their various social identities (as members of the various groups to which they belong; Tajfel & Turner, 1979). Further, it contends that categorizing oneself in terms of a social identity that is shared with others (e.g., as a member of a psychotherapy group) lays the foundation for various group processes, including social influence (Turner, 1991). Social identity leadership theory proposes that leadership is a process of social influence and that any given person's capacity to exert influence (i.e., to lead effectively) rests, at least in part, on their capacity to represent and embody the meaning of the group in a given social context (Haslam et al., 2020; Hogg, 2001). In other words, the theory proposes that any individual group member's ability to exert leadership varies as a function of their *ingroup prototypicality* (referred to simply as prototypicality from hereon).

Empirical research from a range of contexts has supported this proposition. For example, to the extent leaders are perceived as prototypical, research has indicated that (a) they are viewed as more charismatic (Platow et al., 2006), (b) they are more trusted to have the group's best interests at heart (Giessner & van Knippenberg, 2008), (c) they are perceived as more trustworthy in general (Jones et al., 2020), and (d) their actions are perceived as fairer (De Cremer et al., 2010). Additionally, and of particular relevance to the present study, there is also evidence that leaders' perceived prototypicality is positively associated with group members' motivation to pursue group goals (Vogt et al., 2021). In short, research has indicated that leaders (and indeed, all group members) are more influential and effective to the extent that they are perceived as prototypical (see also Steffens et al., 2021 for a recent meta‐analysis).

### What type of prototypicality is most beneficial: aspirational or exemplary?

Despite relatively consistent evidence for the benefits of leaders being perceived as prototypical group members, debate remains regarding the specific way in which leaders should be prototypical. That is, there is conjecture regarding whether it is most beneficial for leaders to represent “who we are” (i.e., our current identity) or “who we want to be” (i.e., our aspired identity). A recent meta‐analysis by Steffens et al. (2021) explored this question and found evidence that, although prototypicality of both forms is a robust predictor of positive outcomes, the aspirational notion of “who we want to be” is more strongly associated with key leadership outcomes (e.g., trust, perceived fairness), than how much leaders represent the average group member. This finding supports theorizing by Abrams et al. (2008) and earlier, Hollander (1958), that it is more important that leaders represent who we aspire to be because this better positions them to effectively lead the group towards a desired future (by directing “us” to achieve important group goals). These researchers suggested that, because a prototypical leader is viewed as working towards the group's best interests, they are granted “innovation credits” (or idiosyncrasy credits for Hollander), which allow them to influence and drive the group towards their vision. Leaders who represent a group's aspired identity may therefore more effectively direct the improvement of a group.

This appears particularly relevant in a group psychotherapy context because (a) the purpose of psychotherapy is to facilitate positive change for participants yet, at the same time, (b) therapists' personal attributes may commonly be perceived to deviate from the typical attributes of their psychotherapy group (“who we are”) on relevant dimensions (e.g., more mentally healthy). These attributes may also, however, commonly represent aspirational goals for group psychotherapy participants (e.g., who typically wish to improve their mental health). It therefore follows that the aspirational notion of prototypicality (“who we want to be”) may be particularly relevant for group psychotherapy leaders and be associated with positive outcomes for psychotherapy participants. More broadly, and from a practical perspective, investigating this is important because, although numerous texts have sought to outline how to effectively lead psychotherapy groups (most notably Yalom & Leszcz, 2020), there is a lack of empirical evidence to support many of the recommendations they contain.

### The present study

The aim of this study was to investigate whether group psychotherapy leaders are more effective to the extent that group members view them as aspirational (i.e., engaging in more positive thoughts and behaviours than group members), compared to exemplary (i.e., engaging in similar thoughts and behaviours to group members). This was explored in a body acceptance group psychotherapy program: The Body Project.

The Body Project is a cognitive behavioural group program designed for young women with body weight or shape concerns that seeks to reduce their risk of developing future eating disorders (Stice et al., 2013). Cruwys et al. (2015b) previously analysed the data upon which the present study draws to examine the program's efficacy. Results also demonstrated that, over the course of therapy, change in perceived group norms (specifically related to the approval of dieting) predicted and preceded improved mental health outcomes. The present study expands on this research by investigating the role that leader prototypicality might play in driving normative change. It is the first empirical study to investigate group psychotherapy leadership from a social identity perspective.

Different forms of group norms were central to our measurement of both leadership effectiveness and prototypicality. In line with the content and goals of the psychotherapy program, the key leadership effectiveness outcome was reductions in approval of dieting (i.e., participants' perceptions of their group's injunctive norms—“what we *should* be doing”; Cialdini et al., 1990). Because a key indicator of prototypicality is the degree to which leaders embody group norms (Hogg & Reid, 2006), prototypicality was conceptualized as participants' perceptions, when they began the program, of how similar they thought group members and leaders were in terms of their frequency of engagement in dieting thoughts and behaviours (i.e., participants' perceptions of the lack of discrepancy between their leader and the group's descriptive norms—“what we *are* doing”; Cialdini et al., 1990).

We hypothesized that, over the course of the psychotherapy program, there would be a greater reduction in the degree to which participants perceived that group members approved of dieting (the desired outcome) to the extent that they perceived their leaders were aspirational at the start of therapy (i.e., perceived to engage in dieting thoughts and behaviours *less often* than group members). In other words, we predicted that leaders would be more effective when participants thought their leaders were aspirational (representing who we *want* to be), compared to when they thought their leaders were exemplars (representing who we *are*).

We also hypothesized that aspirational prototypicality of leaders would predict two therapeutic outcomes (intention to diet and body satisfaction) at the end of therapy, via changes in group approval of dieting across therapy.

## METHOD

### Participants

Participants were drawn from a sample of individuals who responded to advertisements at either a major university or a community health service in Australia inviting girls and women aged 15–25 years experiencing body weight or shape concerns to participate in a Body Acceptance Program. After providing informed consent, eligible respondents were screened using the SCOFF tool (Morgan et al., 1999): “Do you often feel that you cannot control what or how much you eat?” and “Do you often eat, within any two‐hour period, what people would regard as an unusually large amount of food?” Respondents who answered “Yes” to both questions were then asked to complete the Patient Health Questionnaire Eating Disorder Screening Tool (Spitzer et al., 1999). If their responses to this questionnaire indicated that they met the diagnostic criteria for an eating disorder, they were excluded from participation and referred to individual psychological treatment. Respondents were also screened for anorexia nervosa using their body mass index based on self‐reported height and weight, but this did not lead to the exclusion of any participants.

Eligibility criteria were met by 112 young women (*M*_age_ = 18.99; *SD* = 3.12). The majority were white (66.07%), spoke English at home (86.61%) and had a healthy Body Mass Index (75%; *M* = 21.74, *SD* = 3.97). During the study, participants were given the opportunity to complete up to five questionnaires (see below), resulting in a total of 519 observations.

### Procedure

The Body Project group psychotherapy program was run over four one‐hour sessions in accordance with the treatment manual (Stice et al., 2013). In each session, leaders encouraged participants to critique the thin ideal of beauty through verbal, written, and behavioural activities. Thus, leaders aimed to create conditions in which participants would move towards holding healthier body image ideals and norms. Groups consisted of five to eight participants and were co‐led by two facilitators. Facilitators were 16 clinical psychology graduate students (two men, 14 women) who received training and weekly supervision from a clinical psychologist and led between one and three of the 18 therapy groups in varying pairs. The Human Research Ethics committee at an Australian university approved the project (Reference #2013000261).

### Measures

Details of all the measures participants completed during the program are reported elsewhere (Cruwys et al., 2015b). Participants completed extended questionnaires immediately before the first session of the program (T1) and immediately after the final session of the program (T5), and a short one‐page questionnaire immediately after sessions one (T2), two (T3), and three (T4). Measures for T1 were obtained during the first psychotherapy session, after informal discussion and introductions with the group had taken place. Prior to this, participants had all been led through the consenting process by their leaders. Additionally, all participants had met with their group leaders before the groups began to discuss the screening process and their involvement in the study. The T1 questionnaire therefore captured participants' first impressions of the leaders and group members prior to commencing the therapeutic program (and thus prior to any normative change resulting from the program). First impression measures have previously been shown to predict outcomes in group therapy at later time points. For example, Cruwys, Steffens, et al. (2020) found that participants' first impressions of similarity among therapy group members at the start of therapy significantly predicted how connected they felt with that group at the end of therapy (*β* = .31, *p* < .001).

#### Group injunctive norms: approval of dieting

Four items were adapted from various sources (Åstrosm & Rise, 2001; Smith & Louis, 2008; White et al., 2009) to assess participants' perceptions of the approval of dieting among their psychotherapy group members (i.e., their injunctive norms around dieting). This measure was the study's primary dependent variable and has previously been established as a mechanism of change in individual eating disorder risk in the Body Project (Cruwys et al., 2015b). Items included: (1) “If I were to lose weight, members of my Body Acceptance group would…” (1 *Disapprove*–7 *Approve*); (2) “Members of this Body Acceptance Group think that dieting is a good idea” (1 *Strongly Disagree*–7 *Strongly Agree*); (3) “Members of this Body Acceptance group think I…” (1 *Should not lose weight*–7 *Should lose weight*); and (4) “How many members of this Body Acceptance group would think that being thin is a good thing? …” (1 *None of them*–7 *All of them*). The four items were summed to form a composite score (with a possible range of 4–28), where higher scores indicated greater perceived approval of dieting. The scale demonstrated excellent internal consistency across all time points except for T1 (*α*_T1_ = .56, *α*_T2_ = .90, *α*_T3_ = .91, *α*_T4_ = .93, *α*_T5_ = .83). This was likely due to the fourth item being non‐significantly correlated with items one and three at T1 (note that key results are identical if this fourth item is dropped and thus it was retained). However, at every other time point, all items were significantly correlated with each other (mean inter‐item correlation, *r* = .68; range .38–.81).

#### Group descriptive norms: dieting thoughts and behaviours

Participants' perceptions of their group members' engagement in thoughts and behaviours associated with dieting were assessed at baseline (T1) using the six‐item Descriptive Norms for the Pursuit of Thinness Scale (Cruwys et al., 2015a). This measure served as both a covariate (to control for the perceived level of dieting in the group before therapy commenced) and one component of the measure of prototypicality (to calculate a difference score between group members' perceptions of other group members and their leaders' dieting thoughts and behaviours). All items began with “How often do you think members of your Body Acceptance group…”. This was followed by: “go on a diet,” “feel bad about their bodies,” “plan to lose weight,” “wish they were thinner,” “speak negatively about their bodies,” and “feel jealous of thin women.” Items were rated on a seven‐point scale (1 *Never*–7 *Frequently*) and summed to form a composite score (with a possible range of 6–42), where higher scores indicated the thoughts and behaviours were perceived to be more common among group members. Internal consistency was excellent (*α* = .91).

#### Leader dieting thoughts and behaviour

Participants' perceptions of their leaders were also assessed using the same questions about dieting thoughts and behaviours. That is, the prompt “How often do you think your group leaders would…” was followed by the same six items. Participants provided a combined rating for the leadership pair (i.e., they did not rate their two group leaders separately on this measure). Again, the possible range for this measure was 6–42. Internal consistency was also excellent for this measure (*α* = .92).

#### Leader prototypicality

To measure leader prototypicality, the difference was calculated between how often participants thought their fellow group members engaged in dieting thoughts and behaviours (descriptive norms) and a rating of their group leaders on these same behaviours. The rationale was that a large difference between these perceptions (such that group leaders were seen to diet *much less* than group members) indicated that leaders were “aspirational” group members on this dimension. Conversely, if the difference between leaders and group members approached zero, this was thought to indicate that leaders were “exemplars” on this dimension (similar to average group members). We chose to calculate and use a measure of leader prototypicality from T1 because this afforded a test of whether perceptions of leader prototypicality predicted subsequent changes in our dependent variable across therapy.

#### Therapeutic outcomes

Participants' dieting intentions and body satisfaction at T5 were measured as therapeutic outcomes. Dieting intentions were measured using the Dieting Intentions Scale (Cruwys et al., 2013), which includes seven items rated on a seven‐point scale. Two items (“In the next three months, I intend to go on a diet” and “In the next three months, I intend to reduce my calorie intake”) ask participants to rate their agreement (1 *Strongly Disagree*–7 *Strongly Agree*). The remaining five items begin with the prompt “If I diet in the next 3 months, this would be” and ask participants to rate their evaluation of how positive it would be to engage in dieting (e.g., 1 *Harmful*–7 *Beneficial*). Items were summed such that higher scores indicated greater intentions to diet in the next 3 months (possible range 7–49). This scale has been shown to have excellent internal consistency in previous studies (e.g., *α* > .91 in four studies; Cruwys et al., 2013) and in the present study (*α* = .92). Cruwys et al. (2013) also found that scores predicted actual dieting behaviours three months later.

Body satisfaction was measured using the Body Image States Scale (Cash et al., 2002). This scale consists of six items measuring satisfaction with one's body (e.g., shape, weight, attractiveness). All begin with the prompt “Right now I feel,”, followed by nine descriptors that indicate differing levels of that feeling (e.g., 1 *Extremely dissatisfied with my weight*–9 *Extremely satisfied with my weight*). Items are summed such that higher scores indicate greater body satisfaction (possible range 6–54). This scale has been shown to have acceptable internal consistency in prior studies (e.g., *α* = .77 for women; Cash et al., 2002) and in the present study (*α* = .75).

### Analytic approach

Analyses were conducted using *R* (Version 4.04) and the accompanying user interface *RStudio* (Version 1.4.1106) for Windows 10®. R packages used included *lme4* (Bates et al., 2015), *lmerTest* (Kuznetsova et al., 2017), and *sjstats* (Lüdecke, 2021).

#### H1

A mixed‐effects repeated measures analysis was conducted on the focal outcome of perceived group approval of dieting. Observations at each time point (*N* = 519) were nested within participants (*N* = 110), who were nested within psychotherapy groups (*N* = 18). Random intercepts were included for both participant and psychotherapy group. Note that two of the 112 women were not included in the analyses for H1, because they did not answer any of the questions related to any of the predictor measures.

The focal predictor was a two‐way interaction between time and leader prototypicality (i.e., discrepancy between group descriptive dieting norms and the leader). The model also included main effects for time, leader prototypicality, and perceived group descriptive norms about dieting at baseline (to control for how often participants initially thought their fellow group members would diet). All were entered in the model as fixed effects. All variables were standardized before entry into the model. Leader prototypicality type was not split into categories (i.e., aspirational vs. exemplar leaders) but instead conceptualized as a continuum ranging from exemplar to aspirational. It was possible to do this because there were only three negative scores on this leader prototypicality index (−4, −2, −1), indicating a perception that leaders dieted *more* than group members. Scores therefore ranged from close to 0 (exemplar‐type leaders) to 30 (aspirational‐type leaders). Removal of the three participants with negative scores did not affect the results, so they were retained in the analyses.

#### H2

Two mediation models investigated whether prototypicality would predict therapeutic outcomes via changes in group approval of dieting. These tested whether the extent to which leaders were perceived to be aspirationally prototypical at T1 would predict participants' (1) body satisfaction and (2) dieting intentions at T5, via changes in perceived group approval of dieting across therapy (between T1 and T5). All 112 participants were retained in the models (as all had responded to the group approval of dieting and therapeutic outcomes measures).

## RESULTS

### Preliminary analyses

Most participants (71.43%) had no missing data. A significant Little's (1988) Missing Completely at Random test, *χ* ^2^(158) = 224.0, *p* < .001, indicated that data were not missing completely at random. Upon further inspection, it was evident that these patterns were likely due to participants who missed one session also being more likely to miss other sessions. For both the mixed‐effects and mediation models, Full Information Maximum Likelihood was used to manage missing data, ensuring that all observations with data for at least one predictor were included in the final models. The appropriateness of this sophisticated technique for use in longitudinal datasets has been repeatedly demonstrated (Lane, 2008; Larsen, 2011).

A normally distributed residual histogram (and non‐significant Shapiro–Wilk's test, *p* = .087), no significant pattern or clustering in the residual scatterplot, no outliers, and no evidence of multicollinearity also suggested that the assumptions for a mixed‐effects model were met.

### Main analyses

#### H1

The intraclass correlation coefficients in the null model with no predictors suggested that a mixed‐effects approach was appropriate to investigate the effect of leader prototypicality on perceived group approval of dieting over time: 31.6% of the variance in perceived group approval of dieting was accounted for by differences across time points, 21.6% was associated with individual differences between participants, and 2.6% was associated with differences between therapy groups. The primary findings are summarized in Table 1. The final model appeared to be a good fit for the data; it explained 35.9% of the variance in perceived group approval of dieting and was a better fit for the data than both a null model with no predictors, *χ* ^2^(4) = 254.85, *p* < .001, and a model with only main effects, *χ* ^2^(1) = 6.08, *p* = .014.

**TABLE 1:** Mixed‐effect model for perceived group approval of dieting (leadership effectiveness)

Predictor	*β* [95% CI]	*SE*	*df*	*p*
Time	−.37 [−.41, −.32]	.02	410.35	<.001**
Baseline group dieting thoughts and behaviour (covariate)	.30 [.20, .41]	.05	104.17	<.001**
Leader prototypicality	.02 [−.11, .16]	.07	257.53	.712
Time × Leader prototypicality	−.05 [−.09, −.01]	.02	410.08	.014*

*Note*: *p < .05; **p < .01; Model *R* ^2^ = 35.9%.

Abbreviation: CI, confidence interval.

**FIGURE 1:**
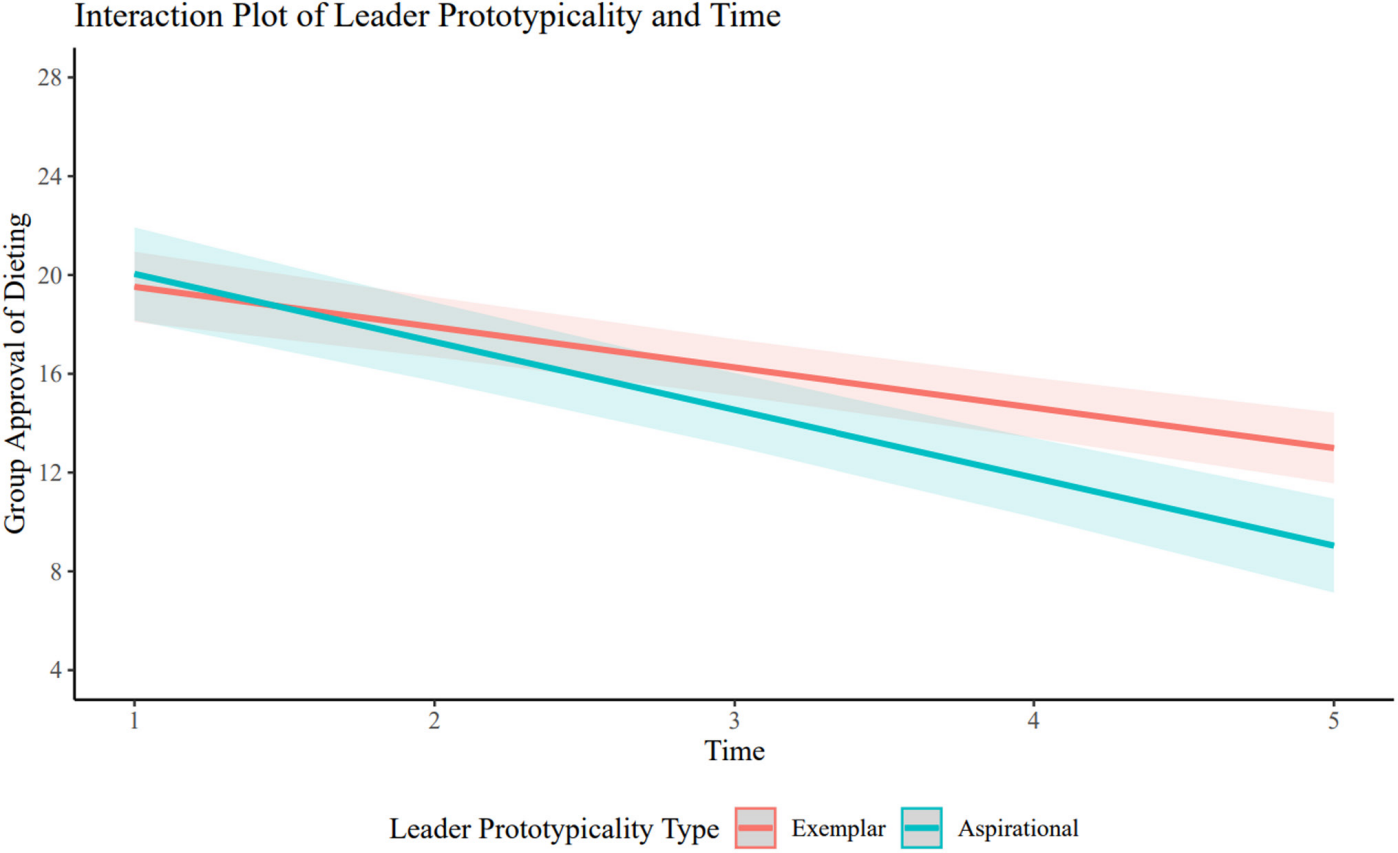
Change in group approval of dieting for different leader prototypicality types. *Note*: This figure plots the pattern of change in group approval of dieting across therapy for two values of our continuous independent variable, leader prototypically. The aspirational notion of prototypicality (i.e., the blue line) represents the *maximum* difference observed between leaders and group members (or a difference score of 30). This predicted greater reductions in approval of dieting over the course of psychotherapy than the exemplary notion of prototypicality (i.e., the red line), which represents the *minimum* difference between leaders and group members (or a difference score of 0).

The main effect of time was significant (*β* = −.37, 95% confidence interval [CI; −.41, −.32], *p* < .001). That is, perceived group approval of dieting significantly decreased over time. The covariate measure of perceived frequency of group dieting at baseline was also significant (*β* = .30, CI [.20, .41], *p* < .001), suggesting that participants who rated their group as engaging in dieting thoughts and behaviours more often at baseline also rated their group as more approving of dieting on average over the course of therapy. The main effect of prototypicality was not significant (*β* = .02, CI [−.11, .16], *p* = .712). In support of the hypothesis, there was a significant interaction between leader prototypicality type and time (*β* = −.05, CI [−.09, −.01], *p* = .014). This interaction was such that the participants' perception of the group's approval of dieting decreased to a greater extent when they thought their leaders were prototypical in the aspirational (rather than exemplary) sense. It should be noted, however, that only an additional .6% of variance in the dependent variable was explained by the model that included this interaction term, compared to the model that did not. Figure 1 shows this interaction; participants who rated their leaders as *most similar* to them (i.e., the exemplar concept of prototypicality; a difference score of 0 between leader and group member frequency of dieting) experienced less change over time than participants who rated their leaders as *least similar* to them (i.e., the aspirational notion of prototypicality; the maximum difference score of 30 between leader and group member frequency of dieting).

#### H2

**FIGURE 2:**
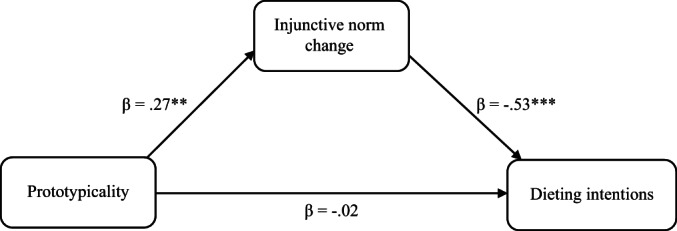
Mediation model examining how prototypicality affected dieting intentions via change in injunctive norms across therapy. The extent to which participants thought their group therapy leaders were aspirationally prototypical predicted participants' reduced dieting intentions via its effect on their perception of their therapy group's approval of dieting (injunctive norms) across therapy. *Note*: Injunctive norm change = change in participant ratings of group injunctive norms surrounding the approval of dieting between the start and end of therapy. Standardized indirect effect of prototypicality on dieting intentions through injunctive norm change: *β* = −.14 95% CI [−.25, −.04], *p* = .007, *SE* = .053. *p < .05; **p < .01; ***p < .001.

**FIGURE 3:**
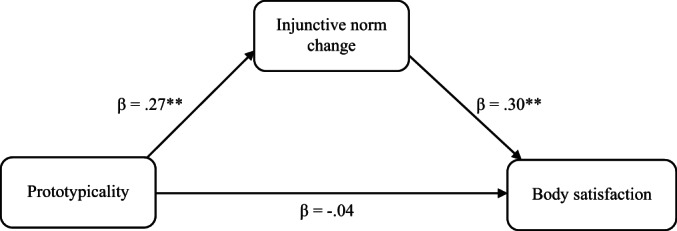
Mediation model examining how prototypicality affected body satisfaction via change in injunctive norms across therapy. The extent to which participants thought their group therapy leaders were aspirationally prototypical predicted participants' increased body satisfaction via its effect on their perception of their therapy group's approval of dieting (injunctive norms) across therapy. *Note*: Injunctive norm change = change in participant ratings of group injunctive norms surrounding the approval of dieting between the start and end of therapy. Standardized indirect effect of prototypicality on body satisfaction through injunctive norm change: *β* = .08 95% CI [.01, .16], *p* = .023, *SE* = .038. *p < .05; **p < .01; ***p < .001.

Significant indirect effects were observed in each of the two mediation models (see Table 2 and Figures 2 and 3 for model details). Prototypicality had a positive indirect effect on body satisfaction ratings (*β* = .08, CI [.01, .16], *p* = .023) and a negative indirect effect on dieting intentions (*β* = −.14, CI [−.25, −.04], *p* = .007). The direct effects between leader prototypicality and both therapeutic outcomes were non‐significant.

**TABLE 2:** Results of mediation models

Clinical outcome	Indirect effects							Direct effects	
	Prototypicality → IN change (*β*)	*p*	IN change → clinical outcome (*β*)	*p*	Indirect effect (*β*[CI])	*p*	*SE*	Prototypicality → clinical outcome (*β*)	*p*
Dieting intentions	.27	.003**	−.53	<.001***	−.14 [−.25, −.04]	.007**	.053	−.02	.768
Body satisfaction	.27	.003**	.30	.001**	.08 [.01, .16]	.023*	.038	−.04	.635

*Note*: IN Change = Change in injunctive norms (perceived group approval of dieting) score between T1 and T5. CI = 95% Confidence Intervals. *N* = 112. Standardized beta coefficients are reported. *p < .05; **p < .01; ***p < .001.

#### Additional analyses

Given that we could also calculate leader prototypicality at T5, we conducted exploratory analyses to investigate how ratings of prototypicality changed across therapy. We calculated the average change in participants' perceptions of how much they thought their leaders versus fellow group members would engage in dieting thoughts and behaviours (i.e., descriptive norms) across therapy (i.e., between T1 and T5). Participants viewed both their fellow group members *and* leaders as less frequently engaging in and thinking about dieting across therapy. Paired samples *t*‐tests confirmed that participants perceived that their fellow group members were dieting significantly more frequently at the start of therapy (*M* = 31.86, *SD* = 6.07) compared to the end of therapy (*M* = 20.51, *SD* = 9.32), *t*(109) = 11.30, *p* < .001. Participants also perceived their leaders to be dieting significantly more frequently at the start (*M* = 19.88, *SD* = 7.85) than at the end of therapy (*M* = 13.03, *SD* = 7.40), *t*(107) = 9.31, *p* < .001. A paired samples *t*‐test also confirmed that the average perceived discrepancy between leaders and group members (i.e., leader prototypicality) reduced significantly between the start (*M* = 11.95, *SD* = 8.05) and the end of therapy (*M* = 7.62, *SD* = 5.55), *t*(106) = 4.63, *p* < .001.

## DISCUSSION

This study examined, for the first time, the role that psychotherapy leaders' prototypicality might play in facilitating positive outcomes among group psychotherapy participants. Addressing an ongoing debate in leadership research (Steffens et al., 2021), it focused on whether it is more beneficial for group psychotherapy leaders to embody the aspirational notion of prototypicality (i.e., “who we want to be”) or the exemplary notion of prototypicality (i.e., “who we are”). In line with H1, results indicated that perceived approval of dieting among psychotherapy group members (an undesirable attitude for participants to hold) decreased to a greater extent when group psychotherapy leaders were perceived as prototypical in an aspirational, rather than exemplary, sense. Additionally, and in line with H2, we found that the extent to which participants perceived their leaders to be aspirational also indirectly predicted important therapeutic outcomes via changes in perceived approval of dieting. Our findings therefore suggest that aspirational leaders may “help us change” in ways that positively impact our mental health.

It should be noted that the effect size for the key effect for H1 (the interaction between leader prototypicality and time) was relatively small. Nevertheless, it is possible that even small changes in perceived group approval of dieting could confer meaningful downstream benefits. Indeed, our analyses for H2 demonstrate this; change in perceived group approval of dieting was a medium to large predictor of two therapeutic outcomes in the mediation models (*β*'s = .30 and −.53). Moreover, several authors have argued that small effect sizes should not be dismissed in psychological research, in part because the effect of focal variables is usually only measured in the short term, and it is possible that these small effects translate into meaningful long‐term consequences (Funder & Ozer, 2019). Additionally, due to the complexities of human cognition, emotion, and behaviour, outcomes in psychology are usually influenced by numerous factors that each have small effects (Götz et al., 2022). In the present study, we found that the way participants viewed their leaders had a small effect on their perception of how acceptable it was to engage in dieting and that this, in turn, had a downstream impact on their mental health (increasing their body satisfaction, reducing their intentions to diet) over a four‐session program. These changes in mental health could have had meaningful future impacts. Our use of a mixed‐effects model may have also affected the accuracy of the effect size estimate. Although there are several benefits of using mixed‐effects models over ordinary least squares regression to analyse hierarchical and longitudinal datasets (e.g., reducing the type‐I error rate; Richter, 2006; Robson & Pevalin, 2016), one downside is that they provide comparatively less accurate effect sizes for individual model terms, such as the effect of an interaction (see Kreft & de Leeuw, 1998; Nezlek, 2001, 2008; Rights & Sterba, 2019). It is therefore possible that the true effect size is larger. The effect size estimate should also be interpreted with some caution.

Given our interest in how group approval of dieting changed across therapy, we also conducted exploratory analyses to determine whether leader prototypicality changed over time. Interestingly, results demonstrated that perceptions of group members and leaders converged across therapy, such that both were perceived to diet less frequently at T5 (with group members perceived to change to a greater extent than leaders). One interpretation for this convergence may be that the leaders became less aspirational over time. Alternatively, the reduced discrepancy between leaders and group members over time may be evidence for the influence of leaders, who led the group to pursue the aspirational example that they had originally set. Further research into how group psychotherapy leaders are perceived over time will enhance understandings of group leadership and provide applied insights into therapy group dynamics.

### Theoretical and practical implications

Our results suggest that group psychotherapy leaders can facilitate positive therapeutic outcomes by portraying themselves as embodying what group members aspire to be. These findings have a range of theoretical and practical implications. First, our findings provide evidence for the applicability of social identity leadership theory to a novel context: group psychotherapy. Building on evidence from research in contexts where leadership has traditionally been examined (e.g., organizations, politics, and sport; e.g., see Cicero et al., 2007; Haslam et al., 2020; Stevens et al., 2021; van Knippenberg & Hogg, 2003), our findings point to the role that group psychotherapy leaders' prototypicality can play in shaping their effectiveness and provide initial evidence that such leaders might most effectively maximize their positive influence by striving to embody who group members want to be, rather than who they are (i.e., to be prototypical in an aspirational rather than exemplary sense). This aligns with findings from a recent meta‐analysis of studies primarily focused on leaders in traditional leadership contexts (e.g., managers in organizations) which found that leaders' perceived aspirational prototypicality was a stronger predictor of positive leadership outcomes than their perceived exemplary prototypicality (Steffens et al., 2021). Thus, our findings demonstrate that the potential added value of aspirational leaders compared to exemplar leaders also applies in the novel context of group psychotherapy.

Second, our results suggest that it may be beneficial for therapists to emphasize that they represent the aspirational identity of the groups that they facilitate. This has implications for the ongoing debate regarding the value of therapists having “lived experience” of the mental health conditions experienced by their clients. On one hand, there is growing empirical support for consumer‐led mental health interventions (Doughty & Tse, 2011; Grey & O'Hagan, 2015). On the other, researchers have raised concerns that consumer‐led healthcare can create role conflict and blur professional boundaries (Faulkner & Basset, 2012)—a perspective supported by evidence that consumer‐led interventions may be less beneficial for severe mental health conditions such as schizophrenia (Lloyd‐Evans et al., 2014). However, when there is specific focus on *recovery*, that is, people with lived experience who have made progress towards the aspirational identity of the group, there is evidence that consumer‐led programs can be effective even for severe mental health conditions. For example, the Hearing Voices Network's consumer‐led support groups focus on recovery, coping, and shared identity and have been demonstrated to be beneficial for people experiencing psychosis (Longden et al., 2018). Our findings raise the possibility that one of the factors that affects whether disclosure of lived experience can enhance therapists' effectiveness may depend, at least in part, on the extent to which the therapist is perceived to embody a future state that group psychotherapy participants aspire towards (e.g., someone who is “in recovery” from their mental health condition).

Along these lines, research has already indicated (a) that connecting with others who are also in recovery is a key healing component of psychotherapy and mutual support groups (Moos, 2007; Rowlands et al., 2021) and (b) that positive therapeutic outcomes flow when individuals internalize new identities that emphasize recovery, rather than maintain mental illness identities (e.g., a person with depression) or identities defined by unhealthy behaviours (e.g., a substance user; Best et al., 2011, 2016; Cruwys & Gunaseelan, 2016; Cruwys, Stewart, et al., 2020; Dingle et al., 2015; McNamara & Parsons, 2016). Notably, Dingle et al. (2015) found that change in identification from a “user identity” to a “recovery identity” in people who attended group psychotherapy for substance use (in a therapeutic community) accounted for substantial variance in positive behaviour change‐up to six months after the end of therapy. In conjunction with this previous research on recovery identities, our findings are consistent with the idea that a leader who shares their personal story of recovery may facilitate positive outcomes, especially if this supports group members to work towards this aspirational representation of the group.

Third, our results may also suggest that, to maximize their influence therapists must not only understand who group members *currently* are, but who they *aspire* to be. However, this is easier said than done, as group norms change dynamically over time. Indeed, albeit outside a therapy context, these ideas have been explored in recent research on dynamic norms (i.e., group norms that are seen to be rapidly increasing or decreasing in a group over time; Loschelder et al., 2019). Sparkman and Walton (2017) found that people will change their behaviour to align with norms that appear to be growing in popularity, even if they (a) contrast what the group is *currently* doing and (b) are only endorsed by a minority of the group. Notably, research also suggests that exposure to dynamic norms may help resolve barriers to personal change by providing evidence that change is possible (Sparkman & Walton, 2019). The concept of dynamic norms may therefore be relevant and useful in the context of group psychotherapy, where leaders are numerically in the minority and seek to change the norms and behaviours of a group. Howe et al. (2021) demonstrated that the influence of dynamic norms on behaviour may be particularly powerful when coupled with appeals that these norms align with collective goals (e.g., that we can “do it together,” p. 215). Therapists might influence collective and dynamic norms by collaboratively setting explicit group goals at the commencement of therapy, as is recommended by most psychotherapy texts (Chen & Rybak, 2004; Sobell & Sobell, 2011; Yalom & Leszcz, 2020).

### Strengths, limitations, and directions for future research

This study had several strengths. These included its high ecological validity, novelty, and strong theoretical underpinning. The longitudinal design was also a strength, as it allowed group member norms to be tracked across a psychotherapy program. Indeed, although this did not afford the same conclusions regarding causality that an experimental study would have, the nature of the design (such that prototypicality was assessed at baseline and normative change was assessed across subsequent time points) increases confidence in the direction of the relationship between the variables we examined.

Despite these strengths, the nature of the research was not able to identify *specific behaviours* that should be pursued by group therapy leaders so as to enhance their aspirational prototypicality. Of course, these behaviours will vary as a function of the goals of the psychotherapy group in question (e.g., improved body acceptance). Nevertheless, future research seeking to identify these behaviours and test their relative efficacy would be beneficial (e.g., see Stevens et al., 2019). Future research would also benefit from (a) assessing the impact of leaders' aspirational prototypicality on a wider range of outcomes, including the extent to which it ultimately leads to better individual health outcomes, and (b) examining other potential mechanisms through which these effects arise. For example, participants' perceptions of therapists' provision of social support may be one particularly promising potential mechanism for investigation (Foran et al., 2021). Future research could reasonably investigate whether aspirationally prototypical group leaders are better able to influence group norms compared to exemplar leaders because they provide support and advice that is more positively interpreted.

## CONCLUSIONS

This study was the first to apply the social identity theory of leadership to a group psychotherapy context. The findings demonstrated that predictions from social identity leadership theory are relevant in a group psychotherapy context; specifically, group leaders may be more effective when they represent the aspirational identity of the psychotherapy group. This can enable them to influence group norms and, through this, facilitate positive therapeutic outcomes for group members.

## AUTHOR CONTRIBUTIONS

**Alysia M. Robertson:** Conceptualization; data curation; formal analysis; methodology; writing – original draft; writing – review and editing. **Tegan Cruwys:** Conceptualization; data curation; investigation; methodology; project administration; supervision; writing – original draft; writing – review and editing. **Mark Stevens:** Conceptualization; supervision; writing – original draft; writing – review and editing. **Michael J. Platow:** Conceptualization; supervision; writing – original draft; writing – review and editing.

## CONFLICT OF INTEREST

The authors declare no conflicts of interest.

## Data Availability

Data are available on request from the authors.
